# High Level of Interaction between Phages and Bacteria in an Artisanal Raw Milk Cheese Microbial Community

**DOI:** 10.1128/msystems.00564-22

**Published:** 2022-12-08

**Authors:** Luciano Lopes Queiroz, Gustavo Augusto Lacorte, William Ricardo Isidorio, Mariza Landgraf, Bernadette Dora Gombossy de Melo Franco, Uelinton Manoel Pinto, Christian Hoffmann

**Affiliations:** a Microbiology Graduate Program, Department of Microbiology, Institute of Biomedical Science, University of São Paulo, São Paulo, São Paulo, Brazil; b Food Research Center, Department of Food Sciences and Experimental Nutrition, Faculty of Pharmaceutical Sciences, University of São Paulo, São Paulo, São Paulo, Brazil; c Instituto Federal de Minas Gerais, Bambuí, Minas Gerais, Brazil; University of Copenhagen

**Keywords:** bacteriophage, Canastra cheese, endogenous starter culture, metagenome, metavirome

## Abstract

Microbial starter cultures are used in the production of many cheeses around the world, such as Parmigiano-Reggiano, in Italy, Époisses, in France, and Canastra, in Brazil, providing many of the unique features of these cheeses. Bacteriophages (phages) are ubiquitous and well known to modulate the structure of bacterial communities, and recent data indicate that cheeses contain a high abundance of naturally occurring phages. Here, we analyze the viral and bacterial metagenomes of Canastra cheese: a traditional artisanal Brazilian cheese produced using an endogenous starter culture and raw milk. Over 1,200 viral operational taxonomic units were recovered using both isolated viral-like particles and complete metagenomic DNA. Common viral families identified included *Siphoviridae* and *Myoviridae*, with 40% of putative phage genomes unidentified at the family level of classification. We observed very high phage diversity, which varied greatly across different cheese producers, with 28% of phage genomes detected in only one producer. Several metagenome-assembled genomes were recovered for lactic acid-producing bacteria, as well as nonstarter bacterial species, and we identified several phage-bacterium interactions, at the strain level of resolution, varying across distinct cheese producers. We postulate that at least one bacterial strain detected could be endogenous and unique to the Canastra cheese-producing region in Brazil and that its growth seems to be modulated by autochthonous phages present in this artisanal production system. This phage-host relationship is likely to influence the fermentation dynamics and ultimately the sensorial profile of these cheeses, with implications for other similar cheese production systems around the world.

**IMPORTANCE** Our work demonstrated a dynamic yet stable microbial ecosystem during cheese production using an endogenous starter culture. This was observed across several distinct producers and was marked by genomic evidence of continued phage-bacterium interactions, such as the presence of bacterial defense mechanisms. Furthermore, we provide evidence of unique microbial signatures for each individual cheese producer studied in the region, a fact that may have profound consequences on product traceability. This was the first effort to describe and understand the bacteriophage composition and ecological dynamics within the Brazilian Canastra cheese production system. The study of this prototypical backslopping production system provides a solid background for further mechanistic studies of the production of many cheeses around the world.

## INTRODUCTION

Viruses are abundant in all ecosystems on Earth, presenting high genetic and taxonomic diversities and shaping biogeochemical cycles and ecosystem dynamics (1, 2). These obligate intracellular parasites are called bacteriophages (or simply phages) when they infect bacteria. The diversity and composition of phage-bacterial communities are influenced by environmental (e.g., pH, temperature, and salinity) and intrinsic biological factors (3, 4). Biological factors can be divided into host traits (host species abundance, organism size, distribution, physiological status, and host range) and virus traits (infection type, virion size, burst size, and latent period) (5).

Many raw milk cheeses around the world are produced using endogenous starter cultures (6), a complex microbial community composed of yeasts, bacteria, and phage, all of which interact to create the final food product. This procedure often uses the backslopping method, where residual fermented whey is collected during production to be reinoculated as a starter culture in the next day’s production batch, effectively creating a continuous microbial growth system (7). Brazil produces a wide variety of artisanal cheeses, several of which use the backslopping method (8, 9). Canastra cheese is one such cheese, and its endogenous starter cultures have recently been characterized using molecular techniques. They contain a diverse but stable microbial community (10). The community stability in these endogenous starters is assumed to be maintained by a continuous diversification process: when one species is excluded, the genetic function is kept present in the environment by another closely related species (11). Studies of the Canastra cheese microbiome are usually focused on the bacterial and fungal components (12, 13); however, little is known about its virome composition and interactions with the bacterial hosts.

Most bacteriophages found in dairy fermentation environments belong to the *Siphoviridae* family, such as the 936 (*Skunavirus*), P335, and c2 (*Ceduovirus*) groups. These nonenveloped viruses possess icosahedral morphology and noncontractile tails, with their genome encoded in double-stranded DNA (dsDNA), and commonly infect bacteria from the *Lactococcus* genus. Other less abundant phage groups are also able to infect bacteria from the genera *Leuconostoc*, *Lactobacillus*, Streptococcus, *Bacillus*, Staphylococcus, and *Listeria* (14, 15). Phage-bacterium interactions tend to be highly specific, due to phage infection strategies that depend on the host binding proteins and the antiphage defense systems in the host (15, 16). These defense systems are classified in adaptive immune systems, including several types of CRISPR-Cas systems, and innate immune systems, such as restriction-modification (RM), abortive infection (Abi) (17), and most recently described, the systems BREX (bacteriophage exclusion) (18) and DISARM (defense island system associated with restriction-modification) (19).

Here, we present the first description of the virome composition in Brazilian artisanal Canastra cheese and the phage-bacterial interactions in this food system. We identified 1,234 viral operational taxonomic units (vOTU) and explored the interactions with bacteria across seven cheese-producing properties using a combination of viral and microbial metagenomic sequencing. We characterized a putative novel species of the Streptococcus phage 987 group, as well as its potential host, a metagenome-assembled genome (MAG) classified as Streptococcus salivarius. Finally, the relationships between 15 complete and high-quality phage genomes and 16 MAGs obtained from starter cultures and cheeses were evaluated.

## RESULTS

### VLP-based description of the bacteriophage community present in the Canastra cheese endogenous starter culture.

We assessed the bacteriophage community present in the endogenous starter cultures used by seven artisanal Canastra cheese producers in Brazil, located in São Roque de Minas and Medeiros, Minas Gerais, Brazil. Viral-like particles (VLPs) were enriched from these starter cultures using a 100-kDa filter membrane and used for metagenome sequencing. The sequencing reads were assembled and rigorously curated to remove bacterial DNA contaminants, producing a final viral sequence catalog. Our final catalog yielded 908 complete and partial viral genomes, with contig sizes ranging from 1 × 10^3^ bp to 1.7 × 10^5^ bp and coverage between 1.41 and 21,108× with genomes classified as complete (*n* = 5), high-quality (*n* = 12), medium-quality (*n* = 23), low-quality (*n* = 584), and not-determined quality (*n* = 284) (see Table S1 in the supplemental material).

10.1128/msystems.00564-22.5 TABLE S1 vOTU table used for analysis. Download Table S1, XLSX file, 0.5 MB. Copyright © 2022 Queiroz et al. 2022 Queiroz et al. https://creativecommons.org/licenses/by/4.0/ This content is distributed under the terms of the Creative Commons Attribution 4.0 International license.

**FIG 1:**
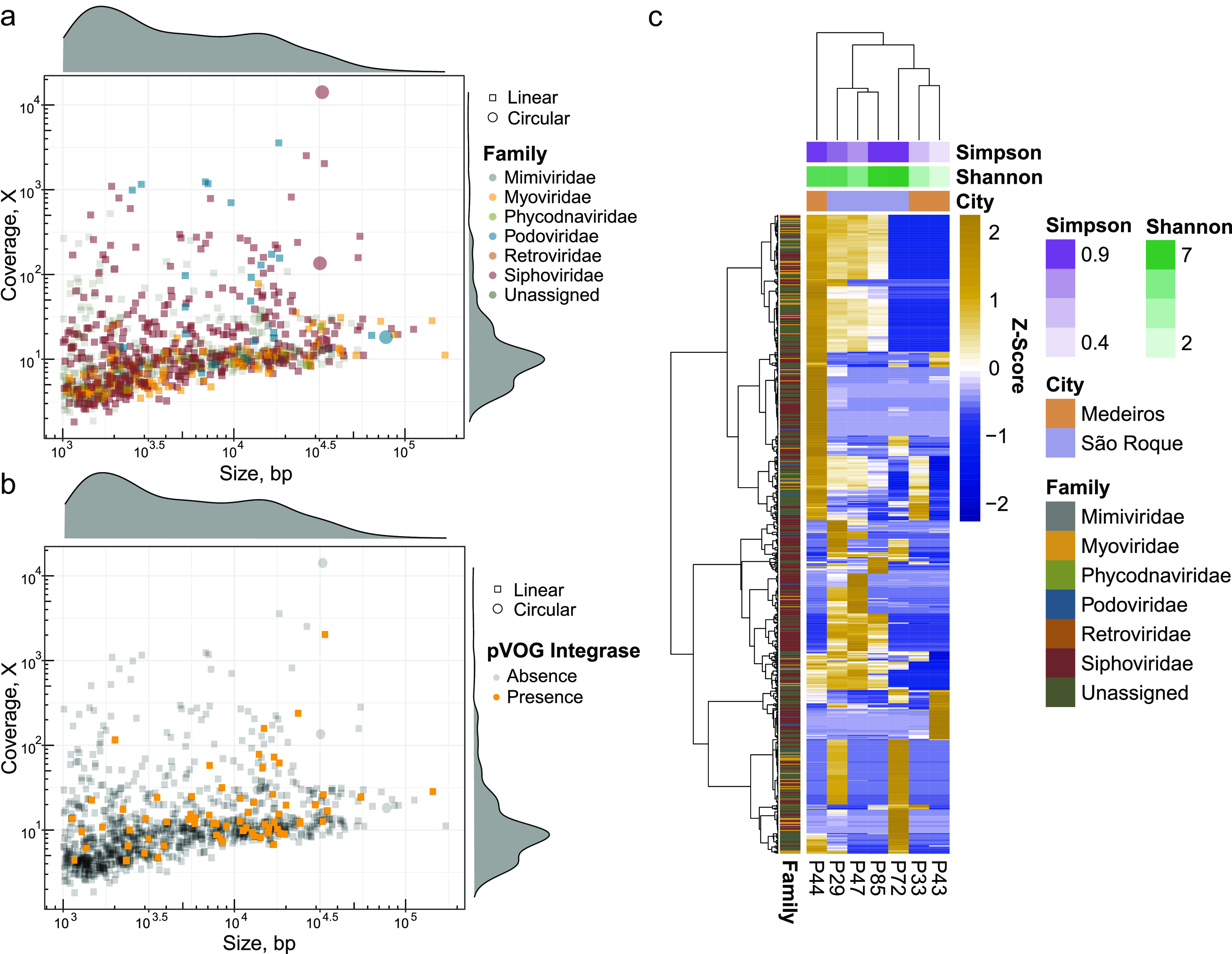
Viral diversity recovered by VLP metagenome sequencing. (a) Size and coverage distribution of the 908 putative viral genomes detected (coverage depth by genome length in bp), color coded by family classification. The families and their proportions were *Siphoviridae* (43.1%), *Myoviridae* (12.1%), *Podoviridae* (3.4%), *Phycodnaviridae* (0.55%), *Mimiviridae* (0.33%), *Retroviridae* (0.11%), and unassigned (40.31%). (b) Temperate phage distribution as detected by the presence of integrase/site-specific recombinase genes. (c) Abundance distribution of viral genomes across starter samples analyzed from 7 distinct cheese producers (viral contig abundances plotted as row “Z-score” using normalized abundance values); only contigs present in at least 2 samples are shown in the heatmap (625 contigs).

Family-level taxonomic classification for the viral catalog was made using the Demovir pipeline. The order *Caudovirales* (99%) prevailed, with only a minor number of sequences classified as *Algavirales* (0.55%) and *Imitervirales* (0.33%) (Table S1). Contigs were classified at the family level as *Siphoviridae* (43.1%), followed by *Myoviridae* (12.1%), *Podoviridae* (3.4%), *Phycodnaviridae* (0.55%), *Mimiviridae* (0.33%), and *Retroviridae* (0.11%). The unassigned contigs corresponded to 40.3% (Fig. 1a). Integrase or site-specific recombinase genes were detected in 67 viral contigs (Fig. 1b), and VirSorter identified 7.38% of this viral sequence catalog as temperate phages (Fig. S2A).

10.1128/msystems.00564-22.2 FIG S2 (a) Viral contigs recovered by viral metagenome analysis (VLPs) and classified as prophages by VirSorter. The size and coverage distribution of 908 putative viral genomes detected (coverage depth by genome length in bp) and which were classified as prophages by VirSorter. (b) Phylogenomic tree of Streptococcus phages. Colors represent the genera of the phages, and the arrow indicates the genomes of phages ph.1.31872 and ph.1.32817 located within the genus 987 group. (c) Compositional profiles of microbial metagenomes in endogenous starter culture and cheese. The relative abundance of each species in 12 paired starter cultures (e.g., P29S) and cheese samples (e.g., P29C) was assessed using MetaPhlAn2. (d) Procrustes analysis using PCoA co-ordinations of starter culture and cheese communities: correlations between viral communities in starter cultures and cheese samples. (e) Procrustes analysis using PCoA co-ordinations of starter culture and cheese communities: correlations between bacterial communities in starter cultures and cheese samples. Distance matrices were calculated using Jensen-Shannon divergence in both cases. Download FIG S2, EPS file, 5.5 MB. Copyright © 2022 Queiroz et al. 2022 Queiroz et al. https://creativecommons.org/licenses/by/4.0/ This content is distributed under the terms of the Creative Commons Attribution 4.0 International license.

Alpha-diversity analysis of viral metagenomes was calculated using a normalized count table of reads mapped against the viral contigs. Four out of seven samples showed high values of diversity index (>6 Shannon and >0.94 Simpson), one sample showed medium values (4.66 Shannon and 0.78 Simpson), and two samples showed low values (<3 Shannon and <0.6 Simpson) (Table S2A). The sample with the lowest diversity values (P43 sample, 1.29 Shannon and 0.39 Simpson) was dominated by one complete, high-coverage viral genome (>21,000×) belonging to the *Siphoviridae* family (Fig. 1c). We observed 28% of phage genomes detected in only one producer, and only 94 contigs were classified at the species level (Table S1; supplementary results at https://doi.org/10.5281/zenodo.7083691).

10.1128/msystems.00564-22.6 TABLE S2 (A) Alpha-diversity of viral metagenomes. (B) Spacers found in MAGs from Canastra cheese microbial metagenomes. (C) Summary of prophages detected in MAGs (intact prophage sequences are in bold). (D) Significant correlations between phage and MAG abundances from starter to cheese. (E) Predicted hosts from 15 complete and high-quality phage genomes and 16 MAGs using WiSH. Significant *P* values based on the null model are presented in bold. Download Table S2, XLSX file, 0.02 MB. Copyright © 2022 Queiroz et al. 2022 Queiroz et al. https://creativecommons.org/licenses/by/4.0/ This content is distributed under the terms of the Creative Commons Attribution 4.0 International license.

### Classification and genome characterization of bacteriophages present in Canastra cheese endogenous starter culture.

**FIG 2:**
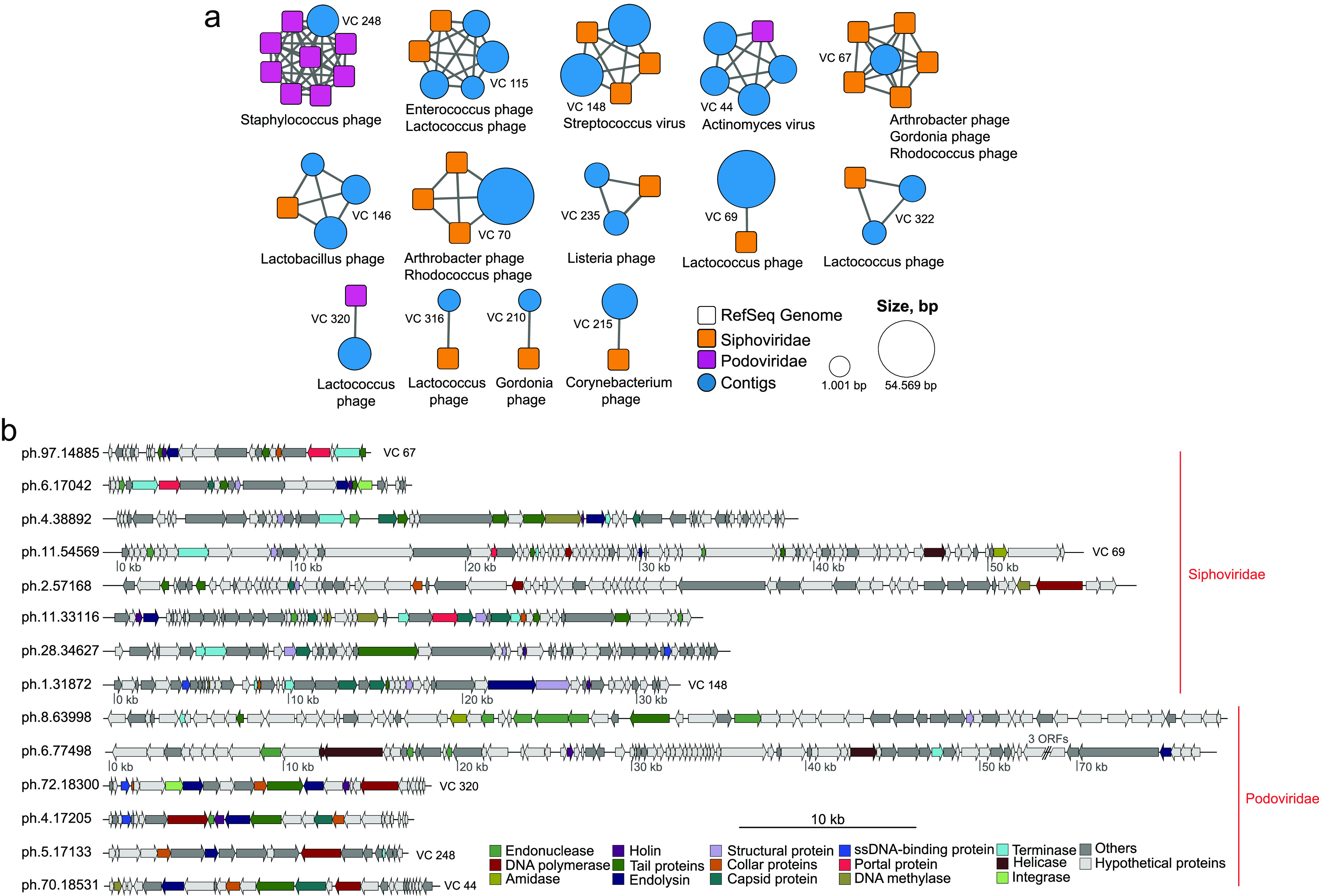
Viral cluster taxonomy and genome annotation. (a) Network of clusters formed between RefSeq genomes and viral contigs. RefSeq genomes and their family level classification are represented by squares, and viral contigs are represented by circles. Square colors indicate viral families, and the size of the circles represents the viral contig length in base pairs. (b) Genome annotation of complete and high-quality bacteriophage genomes using the pVOG database; some of these genomes were clustered with RefSeq genomes as shown by their VC number. Colors indicate the gene identification.

We used the vConTACT v2.0 clustering pipeline to refine the taxonomic assignment of our viral genome catalog (20), using all known viral genomes. Only 2.75% of our contigs had any match against all viral genomes present in RefSeq, indicating a large amount of novel viral diversity (Fig. S1). Viral contigs clustered with RefSeq genomes belong to *Siphoviridae*, *Myoviridae*, and *Podoviridae* families (Fig. 2a). We annotated 14 of our 17 complete and high-quality putative viral genomes using the MultiPhATE2 pipeline (21) and the pVOG database (Fig. 2b). Eight genomes were classified as *Siphoviridae*, with genomes sizes ranging from 14,885 to 57,168 bp, and six as *Podoviridae*, with genomes sizes ranging from 17,133 to 77,498 bp. The most frequently annotated genes in these 14 viral genomes were tail protein (22), terminase (17), endonucleases (15), capsid protein (14), and endolysin (13) (complete annotation is available in Table S3A). We observed integrase genes in two genomes, including a fully recovered genome which clustered with *Lactococcus* phage asccphi28 belonging to the P034 phage species (VC 320).

10.1128/msystems.00564-22.1 FIG S1 Viral clusters (VC) formed between viral contigs. Viral contigs and their family level classification are represented by circles. Colors indicate viral families, and the size of circles represents the viral contig length in base pairs. Download FIG S1, EPS file, 0.8 MB. Copyright © 2022 Queiroz et al. 2022 Queiroz et al. https://creativecommons.org/licenses/by/4.0/ This content is distributed under the terms of the Creative Commons Attribution 4.0 International license.

10.1128/msystems.00564-22.7 TABLE S3 (A) List of genes of complete and high-quality bacteriophage genomes annotated using the pVOG database. (B) ph.1.31872 and ph.1.32817 ORF identity against 9 phages belonging to the phage 987 group. Download Table S3, XLSX file, 0.08 MB. Copyright © 2022 Queiroz et al. 2022 Queiroz et al. https://creativecommons.org/licenses/by/4.0/ This content is distributed under the terms of the Creative Commons Attribution 4.0 International license.

### Novel Streptococcus phages from the 987 group.

**FIG 3:**
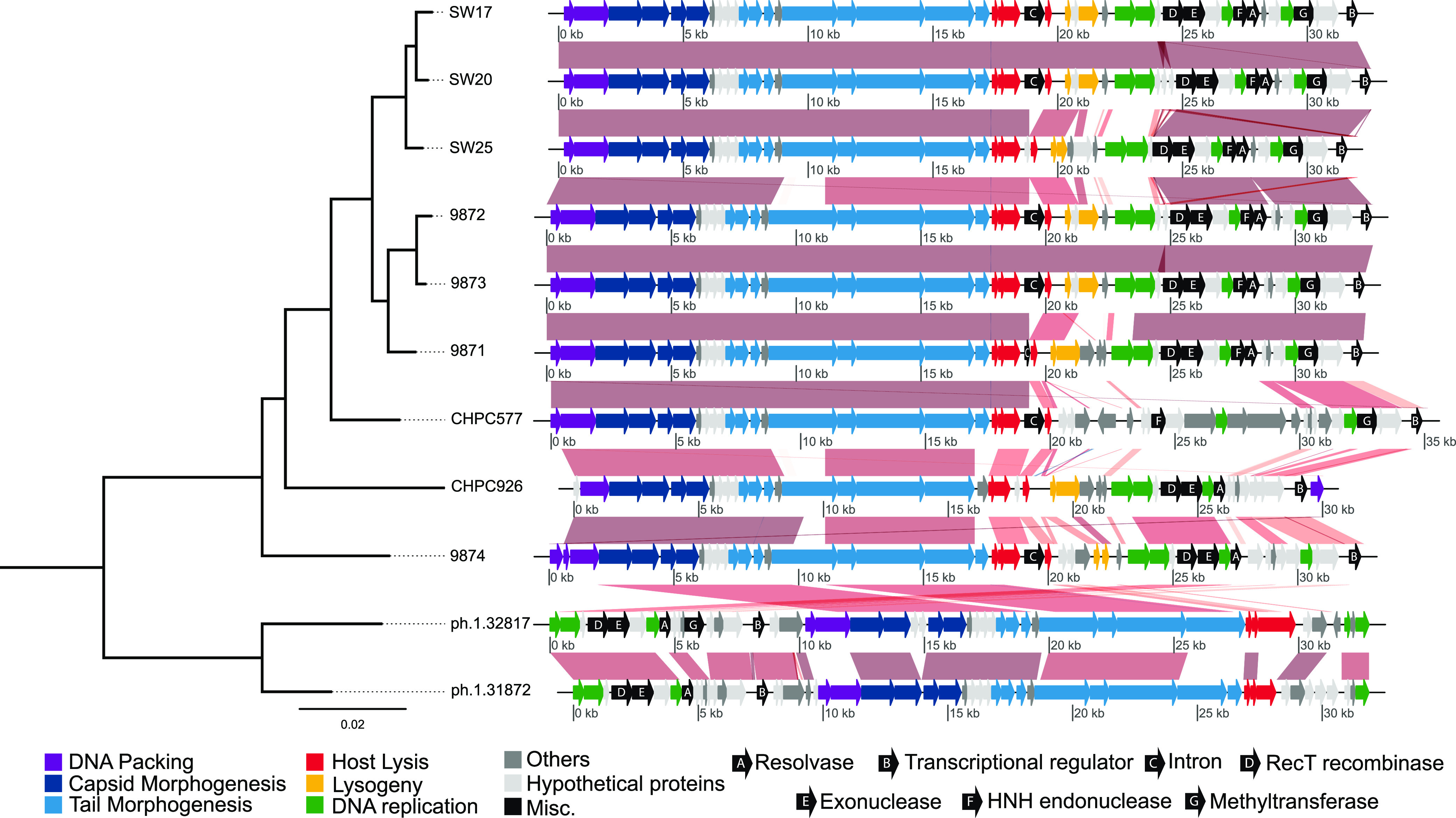
Phylogenomic analysis describing the relationships of 987 group phages previously described and the two novel phages (ph.1.32817 and ph.1.31972) found in our study. Major proteins are color coded as shown; the shaded areas connecting the genomes indicate overall sequence similarity, with darker shades indicating higher similarity. Other notable genes, according to McDonnell et al. (2016) (36), are annotated with arrows to aid in further comparison studies.

Some complete and high-quality genomes were clustered with viral genomes present in RefSeq, such as the novel phages described here, ph.1.31871 and ph.1.31871 (VC148), and Streptococcus *virus* 9871, 9872, and 9874, which belong to the phage 987 group. A phylogenomic analysis of this virus cluster (VC) containing 98 Streptococcus phage genomes placed our novel genomes firmly within the 987 group (Fig. 3 and Fig. S4). Considering that both phages presented <95% average nucleotide identity (ANI) values compared to the four available genomes, we propose that phages ph.1.31871 and ph.1.31871 belong to a novel phage species within this group (Fig. S2b). Although we observed a high similarity (as measured by ANI) between our novel phages ph.1.32817 and ph.1.31872, they did not form a single vOTU, presenting an alignment fraction of less than 85%, further indicating a potential strain differentiation. These two novel 987 group phages also form a separate clade in the phylogenomic analysis and were detected even when we conducted separate assemblies for VLP-derived sequences and for full metagenomics sequences. They present an inversion in the genome structure detected, compared to other phages within the 987 group described in the literature. The most similar open reading frames (ORF) of these novel phages with the other phages in the group are terminase large subunit, RecT recombinase, and methyltransferase (minimum identity within the group of 97.58%, 97.08%, and 96.77%, respectively), and the most diversified ORFs are transcriptional regulator, DNA replication protein, and minor structural protein (minimum identity within the group of 32.77%, 32.95%, and 36%, respectively) (Table S3B).

10.1128/msystems.00564-22.4 FIG S4 Phage-bacterial interaction network during cheese production. Putative phage-bacterial infection interactions for all producers (e.g., P29 and P33) and for separate starter and cheese samples (e.g., P29S and P29C). Nodes represent each MAG and phage analyzed; edges represent putative infection relationships between phage and bacteria. This figure refers to Fig. 5. Download FIG S4, EPS file, 1.6 MB. Copyright © 2022 Queiroz et al. 2022 Queiroz et al. https://creativecommons.org/licenses/by/4.0/ This content is distributed under the terms of the Creative Commons Attribution 4.0 International license.

### Identification of lactic acid bacteria (LAB) in the endogenous starter cultures and cheese samples accessed by microbial metagenome sequencing.

To better understand the microbial community and the interactions between phage and bacteria in Canastra cheese, we also carried out microbial metagenome sequencing using samples of endogenous starters and cheese produced with these same starters at 22-days of ripening, which is the minimal ripening time required by law in Brazil for commercial Canastra cheese (22). These data were initially analyzed using MetaPhlAn2 (23), and the most abundant bacterial species detected were Lactococcus lactis (average of 30.6%), Streptococcus thermophilus (17.4%), Streptococcus infantarius (13.7%), Streptococcus salivarius (7.3%), and Corynebacterium variabile (5.5%) (Fig. S2c). MetaPhlAn2 also detected *Lactococcus* phage ul36 (6.1%) in starter and cheese samples from producer P29 and in the starter from producer P72.

Having established this first characterization of the microbial community, we refined the bacterial species analysis by detecting metagenome-assembled genomes (MAGs). Metagenomic contigs were submitted to the metagenomic workflow of Anvi’o v6.1 (24). Contigs were binned using CONCOCT (25), followed by manual curation. From a total of 50 MAGs obtained, we selected 16 refined MAGs with at least >50% completeness and <10% redundancy, 9 of which showed more than 90% completeness (Table S4A). FastANI (26), CheckM (27), and PhyloPhlAn v3.0 (28) were used to taxonomically classify each of these bins.

10.1128/msystems.00564-22.8 TABLE S4 (A) Taxonomic inference details of 50 MAGs recovered from microbial metagenomes. (B) List of genes classified as antiphage defense mechanisms present in 16 MAGs recovered from Canastra cheese. Download Table S4, XLSX file, 0.04 MB. Copyright © 2022 Queiroz et al. 2022 Queiroz et al. https://creativecommons.org/licenses/by/4.0/ This content is distributed under the terms of the Creative Commons Attribution 4.0 International license.

**FIG 4:**
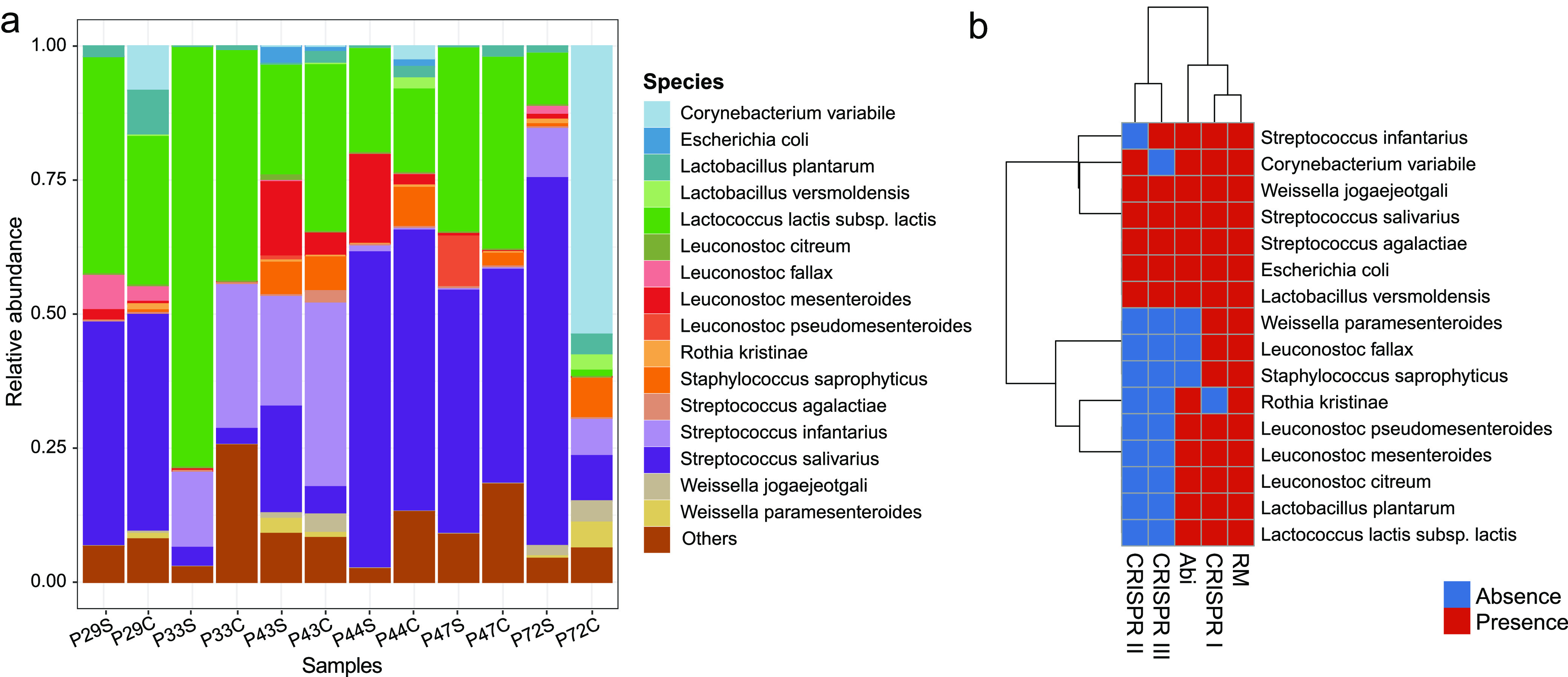
Metagenome-assembled genome (MAG) composition and antiphage defense mechanisms in endogenous starter and cheese. (a) Relative abundance of each MAG classified at the species level in 12 paired starter and cheese samples. “Others” represent reads mapped against lower-quality MAGs. (b) Presence and absence of antiphage defense mechanisms found in each MAG.

We identified 13 MAGs belonging to the *Firmicutes* phylum, two belonging to *Actinobacteria*, and one to *Proteobacteria*. The most representative genera were *Leuconostoc* (4 MAGs), Streptococcus (3 MAGs), and *Lactobacillus* and *Weissella*, (2 MAGs each), all of which had at least 95% average nucleotide identity (ANI) to reference genomes. Other MAGs were classified as *Lactococcus*, *Rothia*, Staphylococcus, *Corynebacterium*, and Escherichia (Fig. 4a; Table S4A). Additionally, MAG07 was assigned as Streptococcus salivarius and showed 94.2% ANI to Streptococcus salivarius BIOML-A24 (GenBank accession no. GCF_009717045.1). A pangenomic analysis carried out with 95 Streptococcus genus reference genomes, and the phylogenomic tree constructed with 302 single-copy core genes, placed *S. salivarius* MAG07 between the *S. salivarius* and S. thermophilus groups, further indicating its potential as a new strain (Fig. S3).

10.1128/msystems.00564-22.3 FIG S3 Pangenomic analysis of the Streptococcus genus. Analysis was performed with 94 complete genomes selected from a previous study and metagenome-assembled genomes classified as Streptococcus salivarius (MAG07; highlighted in purple). Download FIG S3, EPS file, 13.4 MB. Copyright © 2022 Queiroz et al. 2022 Queiroz et al. https://creativecommons.org/licenses/by/4.0/ This content is distributed under the terms of the Creative Commons Attribution 4.0 International license.

The most abundant MAGs across all samples were classified as Streptococcus salivarius, Lactococcus lactis subsp. *lactis*, *and* Streptococcus infantarius (averages of 34.6%, 33%, and 11.4%, respectively), detected in all samples. An inverse relationship was detected between *S. infantarius* and *S. salivarius* abundances across all studied samples. We observed a high level of similarity between the community composition observed in the starter and cheese samples within the same producer (Wilcoxon test *P* value = 0.001, comparison of Bray-Curtis distances within versus between producers), with some species decreasing or increasing in relative abundance, such as the decrease of L. lactis subsp. *lactis* and increase of *S. infantarius* in samples from producer P33. A departure from this tight within-producer relationship between starter and cheese samples was observed only for producer P72, where we observed a marked increase in Corynebacterium variabile relative abundance from starter (0.003%) to cheese (56.9%).

### Antiphage defense mechanisms found in Canastra cheese MAGs.

The 16 MAGs were screened for known bacterial defense system genes and manually filtered for specific antiphage defense mechanisms. We identified a total of 395 defense genes belonging to restriction modification (RM), abortive infection (Abi), and CRISPR-type I, II, and III mechanisms (Fig. 4b). The Rothia kristinae genome had no CRISPR-cas system genes, and all *Leuconostoc* genomes presented only the CRISPR-I system. Weissella jogaejeotgali, Streptococcus salivarius, Streptococcus agalactiae, Escherichia coli, and Lactobacillus versmoldensis harbored all five types of defense genes. Another five MAGs harbored three types of defense genes, such as Lactococcus lactis subsp. *lactis* and Leuconostoc mesenteroides, and four MAGs had only two types of defense genes, Weissella paramesenteroides, Leuconostoc fallax; Staphylococcus saprophyticus harbored RM and CRISPR-type I, and *Rothia kristinae* harbored RM and Abi systems. We did not find any defense genes classified as DISARM, BREX, Thoeris, Shedu, Gabija, and others (Table S4B).

### Presence of CRISPR spacers and prophages in MAGs.

The phage infection history of a given bacterium can be studied by characterizing CRISPR arrays present in their genome. Here, we identified CRISPR spacers in our MAGs using PILER-CR and CRISPRCasFinder (29) (Table S2B); each spacer consensus generated was matched against our viral contig catalog and the IMG/VR database. A total of 10 CRISPR arrays were found in 4 of the 16 MAGs (supplementary data at https://doi.org/10.5281/zenodo.7083691). The MAG classified as Streptococcus salivarius (MAG07) harbored 5 arrays with 153 spacers and an average length of 212 bp. The MAG with the second-most CRISPR arrays was MAG03, classified as Escherichia coli, which harbored 3 arrays with 106 spacers and 267 bp of average length. The other two MAGs (Lactobacillus versmoldensis and Weissella jogaejeotgali) harbored only one array each.

All CRISPR arrays of Streptococcus salivarius matched (more than 95% identity) phages from the IMG/VR database, represented by phages from the families *Siphoviridae* and *Myoviridae* and having the genera Streptococcus and Streptococcus thermophilus as predicted host lineages. The array present in the Weissella jogaejeotgali MAG matched phages from the family *Siphoviridae*, and no match was found for the array present in the Lactobacillus versmoldensis MAG (Table S5). Two of three arrays detected in the E. coli MAG presented sequence signatures for the *Siphoviridae*, *Myoviridae*, *Podoviridae*, and *Inoviridae* (*Tubulavirales*) phage families, all of which have Escherichia, Klebsiella, and *Xanthomonas* species as predicted host lineages. Only one array, detected in the E. coli MAG, matched phage contigs from our viral catalog; one of these was ph.11.54569, classified as a *Lactococcus* phage and grouped within VC69 (Fig. 2). We also identified four intact prophage sequences in our MAGs (Table S2C).

10.1128/msystems.00564-22.9 TABLE S5 Blast results of CRISPR arrays detected in 16 MAGs that matched the IMG/VR database and vOTU table produced in this study. Download Table S5, XLSX file, 0.02 MB. Copyright © 2022 Queiroz et al. 2022 Queiroz et al. https://creativecommons.org/licenses/by/4.0/ This content is distributed under the terms of the Creative Commons Attribution 4.0 International license.

### Phage contig recovery from microbial metagenomes.

It was possible to recover phage sequences not detected in the VLP-isolated data set by analyzing the microbial metagenome data set using VirSorter (30). A total of 514 viral contigs were identified from the 12 metagenome samples, and their taxonomic classification at the family level revealed a prevalence of *Siphoviridae* (85%), followed by *Myoviridae* (4,2%) and *Podoviridae* (2,5%). We recovered 2 complete and 10 high-quality genomes, while the remaining genome fragments were of medium quality (27), low quality (467), and not determined (8). The two complete phage genomes recovered from the bacterial metagenomes were the same as those found when sequencing VLPs obtained from endogenous starter samples of producers P33 and P43 (ph.1.31872 and ph.1.32817).

We expanded our viral catalog to include the new phage detected using the metagenome data set, by comparing all contigs obtained from the viral and bacterial metagenomes using FastANI, creating a final virus list with 1,234 unique phage contigs. These 1,234 contigs were host predicted using CrisprOpenDB (31) (Table S1) and used for comparative analysis of phage and bacterial communities.

### Correlations between phage and MAG populations in cheese metagenomes.

**FIG 5:**
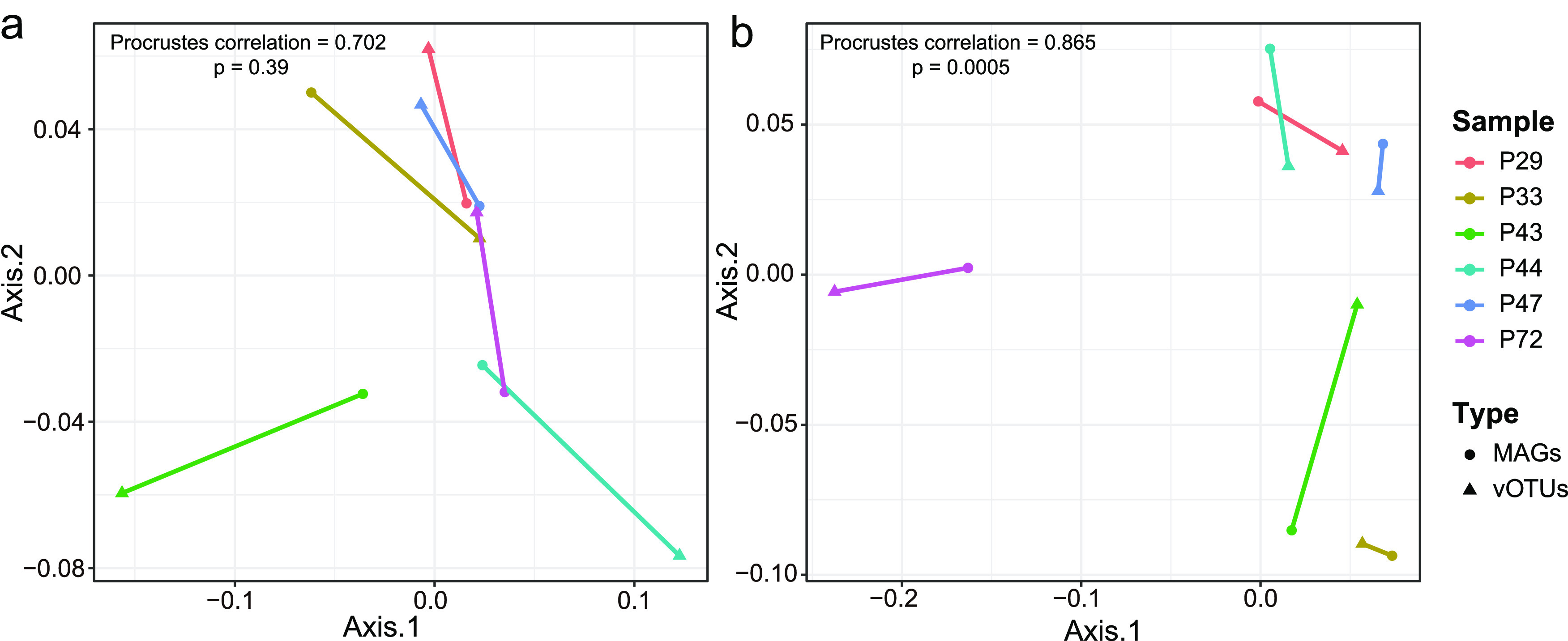
Procrustes analysis using PCoA co-ordinations of viral and bacterial communities. (a) Correlations between viral (vOTUs, *n* = 1,234) and bacterial communities (MAGs, *n* = 16) in endogenous starter culture samples. (b) Correlations between viral and bacterial communities in cheese samples. Distance matrices were calculated using Jensen-Shannon divergence in both cases.

We mapped all reads obtained from each sample to this expanded viral list to create a viral OTU (vOTU) table for further comparative analysis. A Procrustes analysis of Jensen-Shannon divergence matrices did not indicate a correlation between viral and bacterial communities in starter samples (0.702 with *P* = 0.39, Fig. 5a); however, the correlation between the viral and bacterial communities in the cheese samples was significant (0.865 with *P* = 0.0005, Fig. 5b). We also identified a significant correlation between bacterial communities present in starter versus cheese samples, but not for phage communities (Fig. S2d and e).

### Evidence of interactions between phages and bacteria during cheese production.

**FIG 6:**
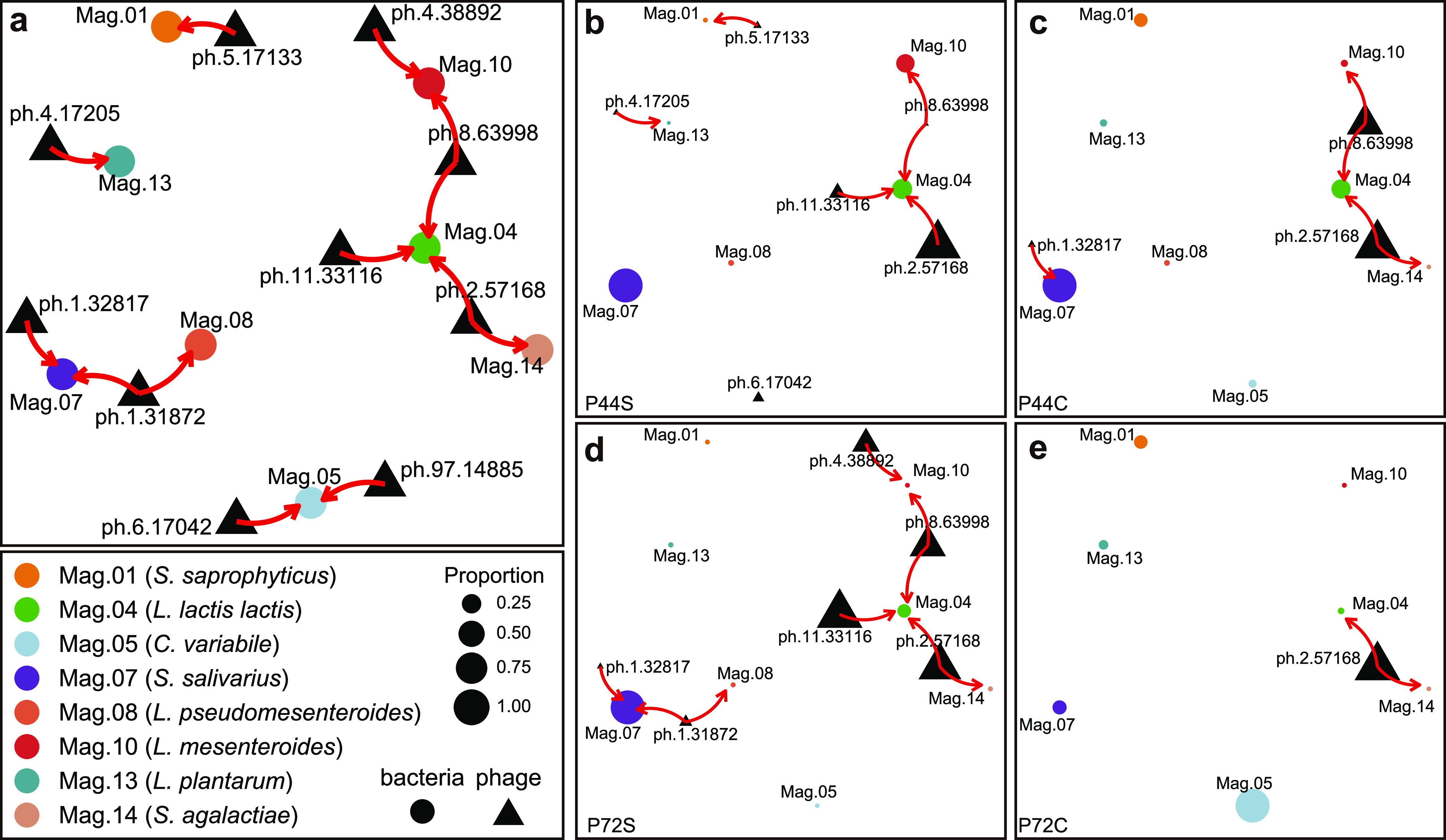
Phage-bacterial interaction network during cheese production. (a) General network describing the putative phage-bacterial infection interactions present across all samples. (b to e) Examples of putative phage-bacterial infection interactions for two distinct producers (P44 [b and c] and P72 [d and e]) and for separate starter and cheese samples (starter [b and d]; cheese [c and e]). Nodes represent each MAG and phage analyzed; edges represent putative infection relationships between phage and bacteria.

We further evaluated the existing relationship between the 15 high-quality phage genomes and 16 high-quality bacterial MAGs in the Canastra cheese production system, using Spearman correlations based on normalized phage/bacterium abundances and on predicted host infection using the software WIsH (32). We considered that phages present in the initial starter culture would infect their hosts during cheese production, and therefore a negative correlation was expected. Indeed, negative correlations were found both in endogenous starters and in cheese samples (Fig. 6 and Table S2D). For instance, phages ph.1.32817 (ρ = −0.84, *P = *0.03) and ph.1.31872 (ρ = −0.88, *P = *0.02) both showed negative correlations with *S. salivarius* (MAG07), and phage ph.1.31872 was also negatively correlated with Leuconostoc pseudomesenteroides (MAG08) (ρ = −0.83, *P = *0.04). We also observed negative correlations between phage ph.11.33116 and L. lactis subsp. *lactis* (MAG04) (ρ = −0.84, *P = *0.03), between phage ph.8.63998 and *L. mesenteroides* (MAG10) (ρ = −0.88, *P = *0.01), and between phage ph.2.57168 and S. agalactiae (MAG14) (ρ = −0.88, *P = *0.01). Additionally, the predicted host for phages ph.8.63998 and ph.2.57168 was L. lactis subsp. *lactis* (MAG04) (null model *P* value < 0.05), and the two phages and MAG04 were present in all samples. Other predicted hosts were *S. saprophyticus* (MAG01) for phage ph.5.17133 and *C. variabile* (MAG05) for phages ph.6.17042 and ph.97.14885 (Table S2E). Using CrisprOpenDB, we could confirm the host prediction to phages ph.1.32817 and ph.1.31872 (Streptococcus) and ph.5.17133 (Staphylococcus). Although an overall correlation pattern could be observed across all samples, we also detected a high level of individual variation across all analyzed producers, indicating a high level of producer specialization (Fig. 6 and Fig. S4).

## DISCUSSION

Endogenous starter cultures are used in the production of several cheeses around the world, such as Parmigiano-Reggiano in Italy, Époisses in France, and Canastra in Brazil. The bacterial composition of these starters is often well characterized, but little is known about their phage-bacterium growth dynamics, with phages normally treated as problems, as viral infections can negatively affect or even eliminate the starter culture during production. Only a few studies characterize the bacteriophage community composition in cheese samples using viral metagenomes, mostly using whey or cheese rind samples (33, 34), and a recent meta-analysis using 184 cheese microbial metagenomes identified a high abundance of phage-associated sequences (35). Here, we report the first study of phage-bacterium dynamics present in an artisanal cheese produced using the backslopping method, as well as its endogenous starter culture, from seven distinct artisanal Canastra cheese producers in Minas Gerais state, Brazil.

We sequenced viral and microbial metagenomes, recovering a total of 1,234 vOTUs, including 18 high-quality or complete viral genomes, and 16 metagenome-assembled bacterial genomes (MAGs). Most of our recovered phage sequences belong to lytic phages, although this could be attributed to the choice of methods used here, which focused on obtaining viral-like particles. The majority of the viral genomes were assigned to the *Siphoviridae*, *Myoviridae*, and *Podoviridae* families, which are commonly encountered in dairy systems (14, 15, 33). We have also observed a high proportion of unclassified contigs or those classified only at the family level, even when they were considered complete and high-quality contigs. There is a high level of viral diversity variation across all analyzed starter samples, with differences as high as 5-fold being observed for the Shannon diversity index. Samples with low viral diversity tended to be dominated by two phages that clustered with Streptococcus virus group 987 reference genomes. This phage group has been recently discovered and described as a novel emerging group of S. thermophilus phages. The group 987 phage is thought to have originated from genetic exchange events between the L. lactis P335 phage group and S. thermophilus phages, acquiring morphogenesis-related genes and replication modules, respectively, from each group (36). We are presenting the first description of the phage 987 group in a Brazilian cheese and the observation of a putative novel phage species in this group. These putative novel species are clustered within a distinct clade in this phage group. They are also marked by an inversion in the phages’ genome structure, highlighting their diversification from previously described members of the 987 group.

One of our most unique findings is the detection of a complete genome, phage ph.72.18300, which clustered with the *Lactococcus* phage asccphi28 genome, belonging to the group P034 phage species (family *Podoviridae*), a group rarely found in the dairy industry (14, 37). *Lactococcus* phage asccphi28 shows more genetic and functional similarities with phages normally infecting Bacillus subtilis and Streptococcus pneumoniae than other Lactococcus lactis phages (37). Our analysis indicates that this phage is potentially a novel viral species within the *Lactococcus* phage asccphi28 group. Another phage genome, ph.5.17133, was classified as a Staphylococcus phage belonging to the genus *Rosenblumvirus* (*Podoviridae*), a phage group currently being considered for its potential use in bacteriophage therapy in veterinary medicine, as a means to treat Staphylococcus-positive mastitis (38, 39).

Recent studies have highlighted the importance of characterizing microbial strains within dairy-related systems to understand microbiome assembly and function in several habitats, such as cheese rinds (35, 40, 41). For instance, distinct bacterial strains can respond to environmental and biological stress in different ways. We have characterized 16 MAGs at strain-level resolution, including LAB such as Lactococcus lactis subsp. *lactis*, Streptococcus salivarius, and Streptococcus infantarius, followed by less abundant strains of *Leuconostoc*, *Lactobacillus*, and *Weissella*. We also found a large amount of evidence for the interaction between phage and bacterial strains in the Canastra cheese production system, with all MAGs showing at least two types of antiphage defense systems, such as CRISPR, restriction-modification (RM), and abortive infection (Abi). The presence of several methyltransferase genes was observed within the phage contigs, which is a well-known phage evasion mechanism against bacterial RM systems (42, 43). Furthermore, we also identified CRISPR spacer arrays in 4 of the 16 analyzed MAGs, indicating previous infections and active evolution of the adaptive immune system in these strains. The MAGs identified as *S. salivarius* and E. coli showed multiple spacers from different phage species, suggesting multiple infection events (44). Additionally, our results demonstrate that Streptococcus salivarius MAG07 is likely a new strain. Therefore, we postulate that the MAG07 Streptococcus salivarius strain could be endogenous to the Canastra region of Brazil. Furthermore, its growth seems to be modulated by native phages present in this artisanal production system, and this relationship is likely to influence the fermentation dynamics and, ultimately, the sensorial profile of these cheeses.

We observed a high level of similarity between proportions of predominant microbial species, from starter to cheese samples, indicating a resilient microbial ecosystem. This also highlights that although microorganisms could be acquired during the cheese production and ripening stages, the overall microbiome composition and structure present in Canastra cheese is primarily determined by the starter culture. Nevertheless, one producer seemed to depart from this pattern in our sampling, where Corynebacterium variabile dominated the cheese samples while being a minor component of the starter culture. *Corynebacterium variable* is found in smear-ripened cheeses and is responsible for flavor and textural properties during the ripening process, and strains of this species are known to be present on the microbiome of cheese surfaces (45). It is possible that *C. variable* is acquired during the maturation process, when the cheese is in direct contact with several surfaces for a prolonged period of time. Overall, our data point to a dynamic host-phage equilibrium in the Canastra cheese production region. While many starter and nonstarter bacteria are likely to be responsible for the overall characteristics observed in cheeses from this region, the growth of distinct bacterial strains could be modulated by specific phage, with a large amount of variation between producers. Such host-phage interactions are likely to be important factors in maintaining the sensorial characteristics of each producer’s cheese.

Phages ph.1.32817 and ph.1.31872 are negatively correlated with *S. salivarius* MAG07, potentially indicating their ability to infect this species. Streptococcus *salivarus* MAG07 was the most abundant Streptococcus species in the absence of 987 phage strains, and when these phages were present, *S. infantarius* MAG06 became the dominant Streptococcus species. Thus, the control of *S. salivarius* by the 987 phage provides an adaptive advantage to *S. infantarius*, allowing it to become the dominant Streptococcus species in this lactic fermentation ecosystem. Phages belonging to the 987 group are also described as being able to infect some *Lactococcus* species; however, we did not observe any evidence for this interaction in our analysis.

For cheese samples, it was possible to detect a global relationship between the composition of phages and bacteria. Biochemical and environmental changes occurring during cheese production and ripening, such as pH and salinity, are known to affect microbiome competition in many types of cheese (6, 8). Thus, it is likely that these same factors could influence phage-bacterial interactions. Other factors intrinsic to lactic fermentation systems, and that can influence phage-bacterial interactions, are the metabolism of residual lactose, lactate, and citrate and lipolysis and proteolysis (46, 47). Previous studies have demonstrated that there is a balance between active starter cells and lysed cells to control the lactose degradation and proteolysis, respectively, and some milk proteins can interfere with phage activity (48, 49). The matrix structure of cheese can also impact those interactions by the firmness and viscosity of cheese (49). Finally, phage dispersion rates in a matrix of hard cheese will be different from those in soft cheese or in starter culture and whey solutions.

In conclusion, this study revealed a rich and diverse phage population permeating all cheese and endogenous starter culture samples, with high levels of phage-bacterial interactions in the Canastra cheese production system. We extensively described the main phage members of these microbial ecosystems and identified a likely novel phage species, belonging to the Streptococcus phage 987 group, as well as its putative host, a novel strain of *S. salivarius*. We observed a dynamic yet stable microbial ecosystem during cheese production, marked by genomic evidence of continued phage-bacterial interactions, where general patterns emerged, yet maintaining a high level of interproducer variability. This is a first effort to describe and understand the viral composition and ecological dynamics within the Canastra cheese production system. We provide a solid background for further mechanistic studies focused on identifying phage-bacterial interactions in artisanal cheeses, with a likely impact in similar production systems around the world.

## MATERIALS AND METHODS

### Sampling.

Samples were collected from seven cheese producers located in São Roque de Minas and Medeiros cities, in the Serra da Canastra region, state of Minas Gerais, Brazil. For the viral metagenome analysis, 50 mL of the endogenous starter culture intended to be used in the daily production and originally obtained from the previous day of production (backslopping method), was sampled and aliquoted in sterile polypropylene tubes. Cheeses produced with these starters were also sampled, at 22-days of ripening, when the cheeses are initially released for sale and human consumption. All samples were placed at −20°C for the duration of the transport (as long as 48h) and shipped frozen overnight to the laboratory, where they were stored at −80°C until further processing.

### Virus-like particle (VLP) concentration.

VLPs were obtained by filtering and centrifugation. Briefly, 50 mL of starter culture was vortexed for 60 s and centrifuged at 2,500 × *g* for 10 min. The supernatant was transferred to a new tube, and the pH was adjusted to ~4.6 with 1 M HCl or 1 M NaOH as needed (33). Adjusted samples were sequentially filtered using 0.45- and 0.22-μm Millex-HV polyvinylidene difluoride (PVDF) syringe filters (Merck Millipore, Tullagreen, Cork, Ireland). Filtered samples were centrifuged at 5,000 × *g* using an Amicon Ultra 15 instrument (100 kDa) (Merck Millipore) following the manufacturer’s protocol (30 min of centrifugation for every 12 mL). The final concentrated solution was diluted on the Amicon filter with sodium chloride-magnesium sulfate (SM) buffer (NaCl, 100 mM; MgSO4·7H20 8 mM; Tris-Cl, 50 mM, pH 7.5), and centrifuged at 5,000 × *g* up until ~3 mL of the concentrated phage solution was recovered (complete protocols are available in supplementary information at https://doi.org/10.5281/zenodo.7083691).

### DNA extraction.

The concentrated VLP stocks were pH-adjusted to 7.5 using HCl of NaOH, if needed, prior to DNA extraction. VLP DNA extraction was made following the protocol developed by Jakočiūnė and Moodley (50) with the DNeasy blood and tissue kit (Qiagen, Inc.). Briefly, 450 μL of concentrated phages was incubated with 50 μL of DNase I 10× buffer, 1 μL of DNase I (1 U/μL), and 1 μL of RNase A (10 mg/mL) (Sigma-Aldrich, St. Louis, MO, USA) for 1.5 h at 37°C. DNase and RNase were inactivated with 20 μL of EDTA (0.5 M) (Sigma-Aldrich) (final concentration of 10 mM) for 20 min at room temperature. To digest the phage protein capsid, 1.25 μL of proteinase K (20 mg/mL) (Invitrogen, Waltham, MA, USA) was added and incubated for 1.5 h at 56°C without agitation. DNA purification was carried out using the DNeasy blood and tissue kit with 500 μL of lysed phage to increase the yield of extracted DNA.

Total DNA was extracted using the E.Z.N.A. soil DNA kit (Omega Bio-Tek, Inc., Norcross, GA, USA). Rind cheese (250 mg) was used for the DNA extraction; 3 mL of starter culture was centrifuged, and the cell pellet was used for extraction according to manufacturer’s instructions. The integrity of the extracted DNA was evaluated by electrophoresis. DNA was quantified using Quant-iT PicoGreen dsDNA assay kit (Thermo Scientific, Waltham, MA, USA) and Synergy H1 hybrid reader (Bio-Tek, Winooski, VT, USA).

### Sequencing.

The Nextera DNA Flex library prep kit (Illumina, San Diego, CA, USA) was used to generate dual-indexed paired-end Illumina sequencing libraries following the manufacturer’s instructions. Libraries were sequenced using 2 × 150-nucleotide (nt) paired-end sequencing runs (4 lanes on separate runs) on a NextSeq genome sequencer (Illumina) with a NextSeq 500/550 high-output kit v2.5 at the Core Facility for Scientific Research, University of Sao Paulo (CEFAP-USP).

### Viral metagenome bioinformatics analyses.

The quality of raw sequences was verified using FastQC v0.11.9 (51). NextSeq adapters were removed using BBDuk (BBTools, https://jgi.doe.gov/data-and-tools/bbtools/) with the following parameters: ktrim=r k=23 mink=11 hdist=1. The quality trimming was also done using BBDuk with the parameters qtrim=r trimq=10 minlen=60 ftr=139. To assemble the quality filtered reads of each viral metagenome, we used SPAdes 3.15.0 (52) with metagenomic function (metaspades.py) and automatic parameters of kmer sizes. Generated contigs were filtered to remove short and redundant sequences using the BBMap function dedupe.sh with the parameters minscaf=1000 sort=length minidentity=90 minlengthpercent=90. Open reading frames (ORFs) were predicted using Prodigal v2.6.3 (53) in metagenomic mode. The final catalog of viral contigs was generated using analysis and criteria similar to those of Shkoporov et al. (54), with some adaptations. Briefly, to search for amino acid sequences of predicted proteins, we used a hidden Markov model (HMM) algorithm (hmmscan from HMMER v3.3) against the HMM database of prokaryotic viral orthologous groups (pVOG) (55) considering the significant hit E value threshold of 10^−5^. Ribosomal proteins were searched using Barnnap v0.9 (https://github.com/tseemann/barrnap) with an E value threshold of 10^−6^. Contigs were aligned against the viral section of the NCBI RefSeq database using BLASTn (56) of the BLAST+ package (57) with the following parameters: E value of <10^−10^, covering >90% of the contig length and >50% identity. We also used VirSorter v1.0.6 (30) for criteria to predict viral sequences with its standard built-in database of viral sequences, with the parameter –db 1. Contigs that met at least one of the following criteria were included in the final catalog of viral sequences: (i) VirSorter positive, (ii) BLASTn alignments to viral section of NCBI RefSeq, (iii) minimum of three ORFs producing HMM hits to the pVOG database, and (iv) circularity.

Contigs selected at the filter step (*n* = 908) were given taxonomic assignment using the Demovir script (https://github.com/feargalr/Demovir) with default parameters and the vConTACT v2.0 (20) clustering pipeline, a network-based analytical tool that uses whole-genome gene-sharing profiles and distance-based hierarchical clustering to group viral contigs into virus clusters (VCs). Besides our viral contigs, we also included in the pool known viral genomes (NCBI RefSeq database release 88). Due to the recent change in viral taxonomy, the results presented here are based only on the old taxonomy. Integrase and site-specific recombinase genes were identified in HMM hits to the pVOG annotation of viral contigs. A counting table of viral contigs was generated, mapping unassembled sequences from each library using BBMap with the following parameters: minid=0.99 ambiguous=random. Read counts for contigs with coverage values less than 1× for 75% of the contig length were set to zero (58). The number of sequences mapped on viral contigs was normalized using the DESeq2 package (59). Completeness, contamination, and quality of contigs were assessed using CheckV (60). We selected 14 contigs classified as complete and high-quality genomes and annotated them using the multiPhATE2 pipeline (21), with ORF prediction with Prodigal and gene annotation with hmmscan using the pVOG database.

A phylogenomic tree of Streptococcus phages was constructed based on the previous study of Philippe et al. (61) (Table S6), using VICTOR (62; Virus Classification and Tree Building Online Resource). Briefly, pairwise comparisons of the nucleotide sequences were realized using the genome-BLAST distance phylogeny (GBDP) method (63) with settings recommended for prokaryotic viruses. The intergenomic distances were used to infer a balanced minimum evolution tree with branch support via FastME v2.0 (64), and branch support was inferred from 100 pseudobootstrap replicates each and visualized with FigTree v1.4.4.

10.1128/msystems.00564-22.10 TABLE S6 List of Streptococcus phages and Streptococcus sp. genomes used in this study. Download Table S6, XLSX file, 0.02 MB. Copyright © 2022 Queiroz et al. 2022 Queiroz et al. https://creativecommons.org/licenses/by/4.0/ This content is distributed under the terms of the Creative Commons Attribution 4.0 International license.

### Microbial metagenome bioinformatics analyses.

The quality of raw sequences was verified using FastQC v0.11.9. NextSeq adapters were removed using BBDuk (BBTools) with the following parameters: ktrim=r k=23 mink=11 hdist=1. The quality trimming was also done using BBDuk, with the parameters qtrim=r trimq=10 minlen=100 ftr=140. Compositional profiles of microbial metagenome samples were assessed using MetaPhlAn2 v2.7.5 (23). To assemble the quality-filtered reads of each microbial metagenome we used SPAdes v3.15.0 (52) with metagenomic function (metaspades.py) and automatic parameters of kmer sizes. The generated contigs were filtered to remove short and redundant sequences using the BBMap function dedupe.sh with the parameters minscaf=1000 sort=length minidentity=90 minlengthpercent=90.

We examined the strain-level metagenome-assembled genomes (MAGs) by coassembling quality-filtered sequences using the MEGAHIT assembler (65), with the parameter –presets meta-large, and the generated contigs were filtered using the BBMap function dedupe.sh with the parameters minscaf=1000 sort=length minidentity=90 minlengthpercent=90. The resulting filtered contigs were submitted to the metagenomic workflow using Anvi’o v6.1 (24). Briefly, we created contig databases, mapping sample reads against contigs using Bowtie 2 (66) and converted SAM files to BAM with SAMtools (67); sequence homologs were searched and added to the contig database with the hidden Markov model (HMM) using HMMER (68); genes were annotated functionally using NCBI’s Clusters of Orthologous Groups (69) and taxonomically using Centrifuge (70); we created an Anvi’o profile database with a contig length cutoff of 2,500 bp; contig binning was done using CONCOCT software (25), and the generated bins were refined manually using anvio-refine function. We selected 16 refined MAGs following the criteria of >50% completeness and <10% redundancy (MAG contigs are available in supplementary data at https://doi.org/10.5281/zenodo.7083691). Taxonomic inference of MAGs was done using CheckM (27) and PhyloPhlAn v3.0 (28) with the database SGB.Nov19; after that, all complete sequences of each MAG species from NCBI RefSeq were downloaded and compared with MAG sequences using FastANI (26). A counting table of viral contigs was generated, mapping unassembled sequences from each library using BBMap with the following parameters: minid=0.99 ambiguous=random. The number of sequences mapped on MAG contigs was normalized using the DESeq2 package. Pangenomic analysis of the Streptococcus genus was performed with 94 complete genomes (Table S6) selected from the previous study by Gao et al. (71) and MAG07 using Anvi’o v6.1 with the pangenomic workflow. Briefly, an Anvi’o genome database was created, computing (with flag: –use-ncbi-blast; and parameters: –minbit 0.5 –mcl-inflation 8) and displaying the pangenome.

CRISPR spacers of MAGs were identified using PILER-CR (72) and further confirmed with CRISPRCasFinder (29). The spacers found were matched with our viral contig catalog and the IMG/VR database (https://img.jgi.doe.gov/cgi-bin/vr/main.cgi) using BLASTn of the BLAST+ package with the following parameters: -qcov_hsp_perc 80 -task blastn -dust no -soft_masking_false (73). Matches of >90% sequence identity for the viral contig catalog and >95% identity for the IMG/VR database were considered. The antiphage defense mechanisms of MAGs were detected following the methods shown by Bezuidt et al. (73). Briefly, we screened predicted genes for domain similarity of known defense systems against the conserved domains database (CDD) of clusters of orthologous groups (COGs) and protein families (Pfams) using RPS-BLAST (E value, <10^−2^) (56). The results were manually filtered for the identification of phage-specific defense systems (complete list is available in supplementary methods). Prophages present in MAGs were detected using the PHASTER web server (74).

Phage contigs present in microbial metagenomes were assessed using VirSorter v1.0.6 (30) as criteria to predict viral sequences, with its standard built-in database of viral sequences with the parameter –db 1. We selected contigs classified only in categories 1, 2 (phages), 4, and 5 (prophages) of VirSorter output (*n* = 514). Open reading frames (ORFs) were predicted using Prodigal in metagenomic mode. Those contigs were submitted to the same process as viral metagenome contigs, ORFs were annotated with pVOG, ribosomal proteins were searched using Barnnap, and contigs were aligned against the viral section of the NCBI RefSeq database using BLASTn. In this case, we used VirSorter positive as the only criterion to include in the final catalog of viral contigs from microbial metagenome sequences. The taxonomic assignments of contigs, completeness, contamination, and quality of contigs were also made using Demovir, vConTACT v2.0, and CheckV with the same parameters used to viral metagenome sequences.

Our vOTU table of contigs was created by combining the two viral contig catalogs (viral and microbial metagenome). We compare the average nucleotide identity (ANI) of contigs from viral and bacterial metagenomes, within and between them, using FastANI, with criteria of ANI of ≥95% and minFraction of >85% (75). A counting table of vOTU contigs was generated mapping unassembled sequences from each library using BBMap with the following parameters: minid=0.99 ambiguous=random. Read counts for contigs with coverage values of less than 1× for 75% of the contig length were set to zero (58). The number of sequences mapped on viral contigs was normalized using the DESeq2 package. These 1,234 contigs were host predicted using CrisprOpenDB (31) to confirm the previous analysis.

### Statistical analysis.

All analyses were carried out using the statistical software R (76) and specific packages as follows: we estimated alpha-diversity using Shannon (log base 2) and Simpson diversity indexes and richness using the number of observed viral OTUs for each sample using the vegan (77) and microbiome (78) packages. We compared similarities between samples of viral and microbial metagenomes through principal-coordinate analysis (PCoA) using Jensen-Shannon divergence, followed by Procrustes analysis using the phyloseq (79) package. Correlations between normalized abundance values of vOTUs present in starter culture versus normalized MAG abundances present in the cheese were calculated to explore potential phage-bacterial predation relationships. Correlations were deemed significant if they had a value equal to or lower than −0.8 and a *P* value of equal to or lower than 0.05. Additional phage-bacterial relationships were explored using WiSH (32) with a null model constructed with 148 crAssphage genomes (they were downloaded using ncbi-genome-download from the viral database with the following parameters: -s genbank, -l “all” –taxid 1978007). Network plots were generated using the R packages igraph (80) and ggnetwork. Genome diagram figures and comparisons were prepared using the GenoPlotR (81) package. Other plots were constructed using ggplot2 (82), ggpubr (83), and pheatmap.

### Code availability.

All software used in this manuscript is currently published and available in the appropriate repositories. They are all listed in Materials and Methods, including the settings used.

### Data availability.

Sequencing data are available in the NCBI under BioProject no. PRJNA747701. Supplemental materials (methods and results), contigs of novel 987 group phages, and metagenome-assembled genomes (MAGs) and their CRISPR spacers are provided at Zenodo (https://doi.org/10.5281/zenodo.7083691).
